# Is it time to rethink the MCCQE Part II?

**Published:** 2016-03-31

**Authors:** Taylor Lougheed

## History

Formed in 1912, the Medical Council of Canada (MCC) was given a “legislated mandate to assure patients that their doctors, wherever they are in Canada and whatever their medical specialty, meet the same demanding, consistent standards.”^1^ This was a time of variable medical training practices, and the new Licentiate of the Medical Council of Canada (LMCC) designation awarded to candidates who completed the appropriate requirements represented a step towards a more standardized, higher quality, and safer medical system. Prior to 1954, those awarded the LMCC designation, following successful completion of the MCC Qualifying Exam (MCCQE), were typically awarded a provincial license to practice medicine.^2^ Following 1954, the LMCC required successful completion of the MCCQE, as well as one year of additional training prior to registration.^2^ This ultimately became the basis for the rotating internship year, after which a provincial license would be granted to allow practice as a “General Practitioner” or “GP.” In the 1980s and early 1990s, however, provincial licensing bodies began moving towards requiring a minimum of two years of post-graduate training, and often required certification through one of the national colleges.^3^ This was followed by changes to the LMCC and the introduction of the MCCQE Part II – a simulated clinical examination taken after a minimum of one year of postgraduate residency training.^4^

## Current Situation

The current LMCC comprises two components:

1. MCCQE Part I, is a computerized exam written at the end of medical school. It includes up to 3.5 hours of multiple choice questions, followed by up to 4 hours of short answer questions designed to “assess knowledge, clinical skills and attitudes as outlined by the Medical Council of Canada (MCC) Objectives.”^5^ Canadian Medical Graduates (CMGs) who took the MCCQE Part I for the first time between 2012–2014 had a pass rate of 98–99%.^6^ The cost to write this exam is currently $1005.^5^
2. MCCQE Part II, contested after at least one year of postgraduate residency training, is a simulated clinical exam designed to “assess the knowledge, skills, and attitudes essential for medical licensure in Canada prior to entry into independent clinical practice.”^7^ It was recently expanded to be spread over two days. CMGs who took the MCCQE Part II for the first time between 2012–2014 had a pass rate of 93–96%.^6^ The cost to take this exam is currently $2409.^5^

The updated licensing requirements developed in 2011 by the Federation of Medical Regulatory Authorities of Canada (FMRAC) and called the “Canadian Standard” state what is currently required to practice medicine in Canada:

1. Have a medical degree.
2. Become a Licentiate of the Medical Council of Canada (complete the MCCQE I and II and its requirements).
3. Complete a postgraduate training program (residency) and associated certification.

## Is the MCCQE Part II still relevant?

Is this clinical exam (MCCQE Part II) truly protecting Canadian patients by assuring them “that their doctors, wherever they are in Canada and whatever their medical specialty, meet the same demanding, consistent standards,”^1^ or are they an outdated requirement, a historical artifact?

The reality is that those who pass or fail the MCCQE Part II are equally unable to practice without further achieving certification through the College of Family Physicians of Canada, the Royal College of Physicians and Surgeons of Canada, or the Collège des médecins du Québec - each of which have final certifying exam requirements. What then, is the importance of an exam that no longer imparts actual practice certification? Is it important for a future orthopedic surgeon to be able to provide counselling on birth control, or an ophthalmologist to examine a hip injury?

## Conclusion

The MCCQE Part II was arguably an important part of licensing for independent practice 15–20 years ago, and provided quality assurance to the public. Under the current regulations, it is required that all new physicians (generalists as well as specialists) achieve further certification through one of the national colleges, making it difficult if not impossible to practice as a GP with only a successful MCCQE 2. This being the current reality, it is important to ask what role the MCCQE Part II now plays in the Canadian medical landscape. I humbly suggest: None.
